# Bolder and Brighter? Exploring Correlations Between Personality and Cognitive Abilities Among Individuals Within a Population of Wild Zebrafish, *Danio rerio*

**DOI:** 10.3389/fnbeh.2020.00138

**Published:** 2020-08-12

**Authors:** Danita K. Daniel, Anuradha Bhat

**Affiliations:** Department of Biological Sciences, Indian Institute of Science Education and Research Kolkata, Kolkata, India

**Keywords:** personality traits, boldness, predation risk, exploratory ability, memory, spatial learning

## Abstract

Within populations, individual differences in behavioral and cognitive traits are dependent on the habitat and specific contexts, such as the presence of a predator or other risks. The ability to show variable responses to changing conditions can be of immense survival advantage to organisms. We studied individual differences in specific personality traits, such as boldness, exploration, and spatial ability, and the effect of these traits on learning ability and memory in the presence of a predatory threat, among wild caught zebrafish (*Danio rerio*). Under laboratory conditions, individuals were trained to perform a simple navigation task, and their performance, exploration, boldness traits were measured, along with learning and memory abilities under two contexts (i.e., in the presence and absence of a predator). Our results revealed that fish showed a clear decline in emergence time, exploration time, and feeding latency over trials, indicative of learning, and further tests for memory also showed memory retention. While the presence of a predator increased emergence time and latencies for navigating, indicating declines in boldness and exploration, these were found to be correlated to different personalities among the individuals and dependent on their sex. While females tended to be bolder and learned the spatial task faster, they showed lower memory retention abilities than males. Personality traits were also found to affect cognitive abilities among individuals. In general, the presence of a predator decreased performance latencies. However, bolder individuals were less affected and emerged more quickly from the refuge chamber than shy individuals. Our results point to the complex interplay of ecological context along with inherent correlations across personality traits that decide the overall personality and cognitive responses among individuals even within populations. These findings thus highlight the importance of an inclusive approach that combines personality and cognition studies for understanding variations within populations.

## Introduction

Until recently, behavioral flexibility has been considered an advantageous trait, as it allows an organism to adapt to unexpected changes in its environment (Dingemanse and Réale, 2005). However, despite adaptability being of vital importance, almost all organisms show rigidity in their behavior, rather than plasticity, at least for some traits, and this has given rise to profound studies in the field of animal personality (Réale et al., 2010). We now know that not only do organisms show consistency in certain types of traits but also that most show some variation at an individual level, and this variation remains more or less constant over time and context (Dingemanse et al., 2009). Like other behavioral traits, cognition in most organisms also depends on environmental factors such as habitat complexity and predator presence and has a tendency to show usage dependent decline with aging (Dukas, 2004). Cognitive abilities such as learning and memory in organisms also show individual variation (Rowe and Healy, 2014; Lucon-Xiccato and Bisazza, 2017a). In this study, we used wild zebrafish *Danio rerio* (Hamilton, 1822) to not only characterize personality traits, such as boldness, exploration, and spatial ability, but also examine the effect of such personality traits on learning ability and memory in a spatial task when provided with a food reward.

Studies across many taxa have shown that some traits, such as boldness, exploration, activity, and aggression can remain consistent within individuals (Réale et al., 2007), and these traits are found to be often correlated (Sih et al., 2012). For instance, in zebrafish, individuals that were bolder in terms of foraging and exploration also showed greater aggression in terms of mate monopolization (Roy and Bhat, 2015). Many instances of such behavioral syndromes have also been noted in three-spined sticklebacks (*Gasterosteus aculeatus*), where population differences are governed by environmental conditions in the local habitat, but there are also individual differences within populations (Bell, 2005).

Personality traits for most organisms govern how they would perform in their natural habitat. For instance, black chickadees that are bolder have a greater tendency to explore and take risks and also perform better at learning foraging tasks, which improve their chances of survival in the wild (Guillette et al., 2009). Similarly, studies on greater tits show that bolder individuals are better suited to finding a mate and show increased aggression and territoriality (Carere et al., 2005).

Just like personality, behaviors that have a direct basis in cognitive processes also show consistency, although it might differ over contexts (Guenther and Brust, 2017). Cognitive behaviors are also related in most organisms, and individuals that show greater cognitive ability in one context also show similarly high cognitive abilities in other aspects (Shettleworth, 2001; Dukas, 2004). Cognitive abilities show variation across populations, indicating that natural habitat directly influences an organism’s ability to learn, make decisions, and retain memory, and it has been observed in several fish species that individuals from more complex habitats show enhanced learning abilities, and individuals from less dynamic habitats show better memory (Girvan and Braithwaite, 1998; Braithwaite et al., 2013; Lucon-Xiccato and Bisazza, 2017a).

Both personality and cognition affect how an organism performs in the environment into which it is placed. Bolder personalities tend to perform well in high-risk, high-reward situations, since their higher activity levels and greater willingness to explore generally ensure that they perform well at tasks such as foraging and adaptation to a changing environment (Carere and Locurto, 2011; Griffin et al., 2015; Blight et al., 2016). Since there is a remarkable consistency in personality and cognitive traits, recent studies test whether there is a relationship between the two (Guenther et al., 2014). Experiments on personality traits and cognitive ability using a variety of behavioral assays to measure various aspects, makes it easier to test for any mutual dependency that might exist.

Studies in other species, such as the eastern water skink (*Eulamprus quoyii*), have shown that individuals that are bolder in terms of emergence from a refuge and exploration, in general, also show better cognitive abilities (Carazo et al., 2014). Personality and cognition studies in brook trout have shown that individuals that are more aggressive in terms of food and mate monopolization are better able to perform a cognitive task, such as navigating a maze (White et al., 2017). Studies on three-spined sticklebacks have shown that boldness directly influences use of information by an individual, and bolder fish are more likely to perform better at perceiving and interpreting environmental cues (Harcourt et al., 2010). Other studies on the same species have shown that bolder fish make faster decisions, but unlike in guppies, where faster decisions lead to more errors (Burns and Rodd, 2008), when it is affected by personality, accuracy does not suffer (Mamuneas et al., 2015).

In zebrafish, personality traits such as boldness, aggression, exploration, and neophilia have been characterized and found to be consistent across contexts (Moretz et al., 2007; Roy et al., 2017). Bolder fish tend to emerge more quickly from a refuge, explore a new arena more readily, and also show less fear in the presence of a predator. Zebrafish have also been models for studying cognitive behaviors such as learning and problem solving (Gerlai, 2016), and it has been shown that some individuals consistently display better learning and memory than others, and this varies across populations (Roy et al., 2017).

Since not much is known about the correlation between personality and cognitive ability in wild zebrafish (*Danio rerio*), our study investigates the underlying pattern, if any exists, between the two and, if so, whether that relationship persists for all individuals within a population.

We tested wild caught zebrafish from a habitat with moderate flow, depth, and vegetation cover for boldness and learning ability in a simple maze setup to look for any correlation between the two kinds of traits. We also tested the fish in the presence and absence of a predator to examine how fish were affected by the appearance of a threat and, if some fish were less affected than others, signifying an inherent difference in personality. Thus, overall, our experiments aimed to answer the following questions:

• Do some individuals (bolder fish) emerge more readily from a refuge chamber and explore?
• In a simple maze setup, is there a reduction in the time taken to navigate the maze and reach a food reward over trials?
• Do fish retain memory of the spatial navigation after a 3-days gap in the training?
• Do bolder fish in terms of emergence from a refuge and exploration also show better learning ability and retention of memory?
• Do males and females show any difference in boldness, learning, and memory?
• Is there a difference in the behavior of fish in the presence and absence of a predator and is that difference less pronounced for bold fish?
• Does learning ability have an effect on memory and how threats are perceived by individual fish?

## Materials and Methods

### Collection and Maintenance of Fish

Wild zebrafish were collected from the Leturakhal stream habitat in West Bengal (India). This habitat had a moderate flow regime (0.8 m/s ± 0.25 m/s) and moderately turbid water (TDS = 52 ppm ± 4 ppm) with riparian vegetation along the banks. Fish were caught using drag nets and transported to the laboratory in aerated plastic bags. Information about environmental factors was also collected, which included water quality parameters and stream characteristics – pH, conductivity, total dissolved solids, and water temperature using a Hanna multiparameter HI991300 probe; dissolved oxygen using YSI DO meter (YSI55DO); stream width and stream depth using measurement tapes, taking an average of three measurements at each site; water velocity (by measuring velocity of floating cork); and altitude using GARMIN trex 30. The parameters were found to be as follows – dissolved oxygen (5.61 mg/L ± 2.3 mg/L), conductivity (98 S/m ± 13 S/m), temperature (23°C ± 0.7°C), pH (7.47 ± 0.02). Coexisting fish species were also noted for future reference.

In laboratory conditions, fish were maintained in aerated bare glass tanks (60 × 30 × 30 cm) containing filtered water. Each tank housed between 150 and 200 fish, to maintain an approximately constant density. Water temperature was maintained between 21 and 24°C, and fish were kept in an LD 12:12 hr light-dark cycle. Food, consisting of either loose or compressed freeze-dried blood worms was provided *ad libitum* once a day. Fish were maintained in lab conditions for at least 2 months for acclimatization before being used for experiments.

### Experimental Setup

**FIGURE 1:**
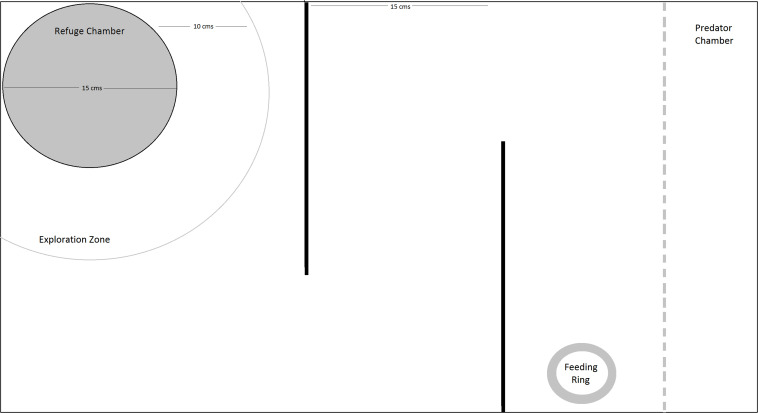
Schematic representation of the top view of the experimental setup.

Fish were trained and tested for learning and memory in a simple maze using a food reward provided in a colored feeding ring (Figure 1). The setup included a refuge chamber with plants, in one corner, which was about 15 cm in diameter, with an 7 × 7 cm opening, to allow the fish to enter the main experimental tank. The refuge chamber was covered and had artificial plants to provide a sheltered environment. An arc roughly five body lengths (12.5 cm) from the refuge chamber was allotted as the exploration zone. The end of the tank opposite to the refuge chamber was designated as the predator compartment and was separated from the rest of the tank by a transparent, perforated plastic barrier. Water level was maintained at 15 cm for all the trials.

### Isolation and Tracking of Experimental Fish

The experimental fish (*n* = 40; 19 males and 21 females) were isolated into tanks with mesh separated compartments 48 h prior to the beginning of the experiment, allowing identification of individuals as well as, communication with each other using visual and olfactory cues across the mesh. This also allowed for tracking individuals across training trials. Mesh-separated compartments were used to minimize any effect of isolation on individuals (Roy et al., 2017). Standard length of each fish was measured after the completion of the experiment (males = 2.43 cm ± 0.22 cm; females = 2.54 cm ± 0.33 cm).

### Training and Testing

Fish were trained for eight trials on consecutive days followed by testing after a 3-day gap on days 12 and 13. For each trial, a single fish was introduced into the refuge chamber and allowed to acclimatize for 2 min, after which the window in the chamber was opened and the fish was allowed to swim out. Once it crossed the exploration zone, food was dropped into the feeding ring in the form of two freeze-dried bloodworms, each about 1 cm in length. Video recordings were carried out using an HD camcorder Canon LEGRIA HF R306, which was placed overlooking the tank, for a maximum of 20 min from the time the food was dropped, and later analyzed to get measures of boldness as well as spatial and cognitive abilities. If the fish failed to emerge until 5 min, or feed until 15 min after the food was dropped for more than 3 days, it was eliminated from the trials and was not tested upon further. Eight fish were thus eliminated from the sample due to death or a lack of performance in the trials.

### Introduction of Predator

The predator used for the experiment was a snakehead (*Channa* spp.), which is a commonly occurring predator in natural habitats with zebrafish populations (Spence et al., 2008). The individual used for the test was approximately 13 cm in length. On day 13, after the first test trial day, it was introduced into the predator compartment and left for an hour to allow it to acclimatize and also for any olfactory cues to be evenly distributed throughout the tank.

### Measures of Personality

On the first day, the time taken by the fish to emerge from the refuge chamber (emergence time) was taken as a measure of boldness. Similarly, time taken to move cross the exploration arc once a fish has emerged was considered the exploration time, which can also be considered another measure of boldness.

The emergence time, exploration time, and overall performance of the fish in the test trial with the predator, and how they differ from the previous test trial are also indicators of boldness of a fish in this arena. Individuals were classified as “bold” and “shy” based on a ranking of the fish by their emergence time on the first day. Of the total individuals (i.e., *n* = 32) tested, half of those with the shortest emergence time on the first day were designated as “bold,” while the other half were designated as “shy.” A similar method was used to classify tested individuals as fast and slow explorers, where ranking was done based on exploration time on the first day. On the first day of training, when a naive fish is introduced into the unfamiliar arena, the time that it takes to traverse the maze and reach the food has been taken as a measure of spatial ability. Fish were grouped into better and worse navigators by ranking the time taken to traverse the maze and feed on day 1, as done with boldness and exploration measures.

### Measures of Cognition

The quantitative improvement in the performance of a fish from the day of the first trial to the last training trial is an indicator of how well a fish has been able to learn and has been calculated as the difference between the feeding latencies on days 8 and 2. The rate of learning was taken as the slope of the regression line for the time taken to feed, for each fish over all eight training trials. The steepness of the slope is a measure of how quickly or slowly the performance of each fish improves over course of the trials. Fish were grouped by ranking both rate of learning as well as improvement in performance and splitting the sample into two. Fish with greater slope of learning were considered to be “fast learners” as opposed to “slow learners,” who had lesser slope in their learning curves. Fish that showed greater improvement in performance were considered “better learners” as compared to fish that showed less improvement in performance and were typed as “poorer learners.”

Memory was measured by how well the fish performed in the test trial after the 3-day gap, taken as the difference in the time taken to reach the food on the test trial and the last training trial.

### Predator Influence on Measures

The effect of the predator was measured by the difference in emergence time, exploration time, and feeding latency between the test trials in the presence and absence of the snakehead. The difference in the time taken to emerge and explore are indicators of how behavior in general is affected, whereas a difference in the feeding latency would point toward the performance being affected by the presence of the predator.

### Ethics Statement

The study complied with the existing rules and guidelines outlined by the Committee for the Purpose of Control and Supervision of Experiments on Animals (CPCSEA), Government of India, the Institutional Animal Ethics Committee’s (IAEC), and guidelines of the Indian Institute of Science Education and Research (IISER) Kolkata. All experimental protocols followed here have been approved by IAEC and guidelines of IISER Kolkata, Government of India. No animals were euthanized or sacrificed during any part of the study, and behavioral observations were conducted without any chemical treatment on the individuals. At the end of the experiments, all fish were returned to stock tanks and continued to be maintained in the laboratory.

### Statistical Analysis

All statistical analyses were conducted in R Studio (ver. 3.6.0, R Core Team, 2019). Distribution of data was tested using package “*fitdistrplus*” (Delignette-Muller and Dutang, 2015). As the data were found to be non-normally distributed, non-parametric tests were used for analysis.

Generalized linear mixed models (GLMMs) using penalized quasi-likelihood (GLMM PQL) (Schall, 1991; Wolfinger and O’Connell, 1993) were built to test the effect of sex, body size, and trial on boldness, exploration, and feeding latency. Fish ID was taken as the random factor and sex, size, and trial were taken as the fixed factors for each of the dependent (predictor) variables – emergence time, exploration time, and feeding time. All GLMMs were built using “*MASS*” package (Venables and Ripley, 2002). The effect of boldness, exploration, and spatial ability on each other and on the measures of learning, memory, and predator influence were tested using correlation tests. Correlations between various behavioral responses were tested using the Spearman’s rank correlation.

Fish were divided into two groups based on sex, or their measures for boldness, exploration, and spatial ability, and the groups were compared to each other. Non-parametric unpaired and paired comparisons (i.e., Mann–Whitney U and Wilcoxon-signed rank tests) were used for all pairwise comparisons as suitable for paired and unpaired samples. The “*MASS*” package was also used to perform the rest of the analyses to test correlation between measures, as well as paired and unpaired comparisons using Wilcoxon and Mann–Whitney *U*-test. Scatter plots for correlations were obtained using the package “*ggpubr*” (Kassambara, 2020), and boxplots were obtained using “*ggplot2*” (Wickham, 2016).

## Results

The selected models for emergence time (Table 1), exploration time (Table 2), and feeding time (Table 3) showed a clear effect of sex and trial for all measures. Details on the mean values of each measured behavioral trait can be seen in the Online Resource (Supplementary Tables S1, S2). There was significant reduction in emergence time (*W* = 528, *p* < 0.001, *n* = 32), exploration time (*W* = 510, *p* < 0.001, *n* = 32), and feeding latency (*W* = 528, *p* < 0.001, *n* = 32) from trial 2 to 8. There was also a difference in emergence time (*W* = 49, *p* < 0.001, *n* = 32) as well performance in the spatial task (*W* = 0, *p* < 0.001, *n* = 32) between trial 8 and the test trial, with both increasing after the 3-day gap, but there was no effect on the exploration time (*W* = 242.5, *p* = 0.37, *n* = 32) (Supplementary Figure S1).

**TABLE 1:** Selected generalized linear mixed models using penalized quasi-likelihood (GLMM PQL) for emergence time, showing the effect of sex and trial.

Emergence time ∼ Sex + Trial					

	Value	Std. Error	DF	*t*-value	*p*-value
Intercept	6.44	0.14	191	45.62	0.000
Sex M	–0.38	0.16	30	–2.34	0.026
Trial	–0.45	0.016	191	–27.94	0.000

*Estimate values, standard errors, degrees of freedom (DF), t-values, and p-values are shown.*

**TABLE 2:** Selected generalized linear mixed models using penalized quasi-likelihood (GLMM PQL) for exploration time, showing the effect of sex and trial.

Exploration time ∼ Sex + Trial					

	Value	Std. Error	DF	*t*-value	*p*-value
Intercept	2.80	0.10	191	25.63	0.000
Sex M	–0.35	0.09	30	–3.59	0.001
Trial	–0.11	0.01	191	6.61	0.000

*Estimate values, standard errors, degrees of freedom (DF), t-values, and p-values are shown.*

**TABLE 3:** Selected generalized linear mixed models using penalized quasi-likelihood (GLMM PQL) model for feeding time, showing the effect of sex and trial.

Feeding time ∼ Sex + Trial					

	Value	Std. Error	DF	*t*−value	*p*-value
Intercept	7.81	0.05	191	143.37	0e+00
Sex M	–0.18	0.04	30	–4.20	2e−04
Trial	–0.47	0.01	191	–52.44	0e+00

*Estimate values, standard errors, degrees of freedom (DF), t-values, and p-values are shown.*

### Effect of Boldness

**FIGURE 2:**
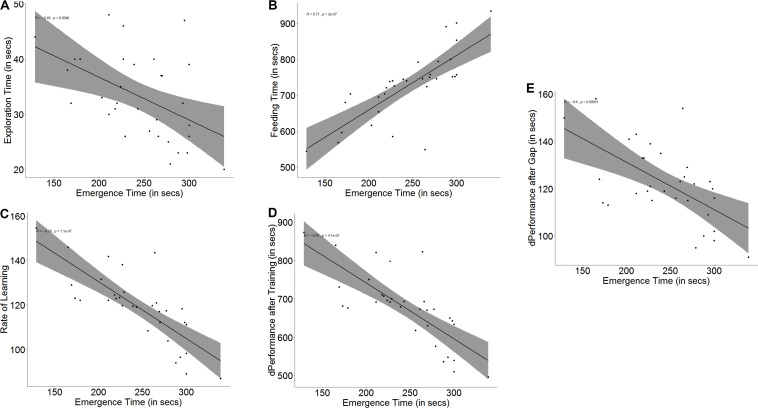
Scatter plot with regression line showing correlation between **(A)** emergence time and exploration, **(B)** feeding time, **(C)** slope of learning curve, which is indicative of the rate of learning, **(D)** difference in performance after training, and **(E)** difference in performance after a 3-days gap.

Bolder fish took less time to emerge from the refuge chamber on the first day of the trials than shy fish (*U* = 0, *p* < 0.001, *n*_1_ = 16, *n*_2_ = 16). Boldness had an effect on exploration (*r* = -0.49, *p* = 0.005, Figure 2A) as well as spatial ability (*r* = 0.77, *p* < 0.001, Figure 2B) in trial 1. Bold and shy fish differed in the time taken to explore the arena (*U* = 195, *p* = 0.012, *n*_1_ = 16, *n*_2_ = 16) and to navigate and reach the end of the maze (*U* = 20, *p* < 0.001, *n*_1_ = 16, *n*_2_ = 16).

Both measures of a fish’s ability to learn, namely improvement in performance (*r* = -0.76, *p* < 0.001, Figure 2D) and learning rate (*r* = -0.78, *p* < 0.001, Figure 2C) depended on boldness. Not only did bolder fish show a greater improvement in their performance (*U* = 236.5, *p* < 0.001, *n*_1_ = 16, *n*_2_ = 16) at the end of the training trials, showing that they learned better, but they also showed a steeper learning curve (*U* = 233, *p* < 0.001, *n*_1_ = 16, *n*_2_ = 16), signifying that they learnt much faster than their shyer conspecifics. Memory in terms of retention also depends on boldness (*r* = -0.60, *p* < 0.001, Figure 2E). Bolder fish showed greater difference in the time taken to navigate the maze and reach the food between the last training trial and the first test trial after a 3-day gap (*U* = 189.5, *p* = 0.021, *n*_1_ = 16, *n*_2_ = 16) (see Supplementary Figure S2).

### Effect of Exploration Tendency

**FIGURE 3:**
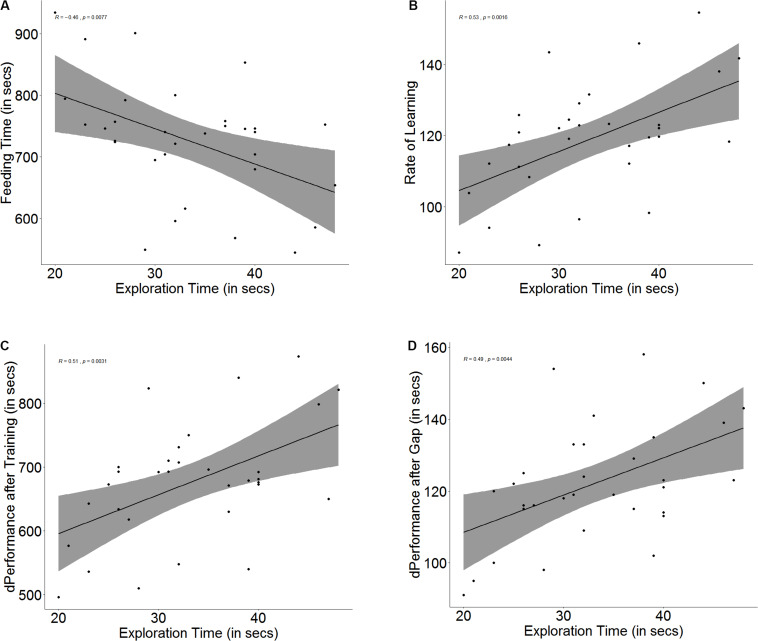
Scatter plot with regression line showing correlation between **(A)** exploration time and feeding time, **(B)** slope of learning curve, indicative of rate of learning, **(C)** difference in performance after training, and **(D)** difference in performance after a 3-days gap.

There is an innate difference in exploration tendency, and some fish tend to explore more readily than others (*U* = 1, *p* < 0.001, *n*_1_ = 16, *n*_2_ = 16), as they take less time to cross the exploration zone. Although a correlation was found between the time taken to explore and the time taken to navigate the maze on the day of the baseline trial (*r* = -0.46, *p* = 0.007, Figure 3A), the difference in feeding time is not significant between fish that are more ready to explore than others (*U* = 158, *p* = 0.27, *n*_1_ = 16, *n*_2_ = 16).

There was a significant correlation between exploration and learning rate (*r* = 0.53, *p* = 0.002, Figure 3B) and quality (*r* = 0.50, *p* = 0.003, Figure 3C); however, fast explorers did not show steeper learning curves (*U* = 90, *p* = 0.16, *n*_1_ = 16, *n*_2_ = 16) or greater difference in performance after training (*U* = 103, *p* = 0.36, *n*_1_ = 16, *n*_2_ = 16) than slow explorers. There was also a significant correlation between exploration time and the difference in performance between the last training trial and the test trial (*r* = 0.49, *p* = 0.004, Figure 3D), but there was no difference in the retention between fast and slow explorers (*U* = 91, *p* = 0.17, *n*_1_ = 16, *n*_2_ = 16) (see Supplementary Figure S3).

### Effect of Spatial Navigation Ability

Some fish that were able to navigate the maze faster on the first day of the experiment reached the food faster and thus were considered to have better spatial navigation ability than those that took longer to reach the reward (*U* = 0.5, *p* < 0.001, *n*_1_ = 16, *n*_2_ = 16).

**FIGURE 4:**
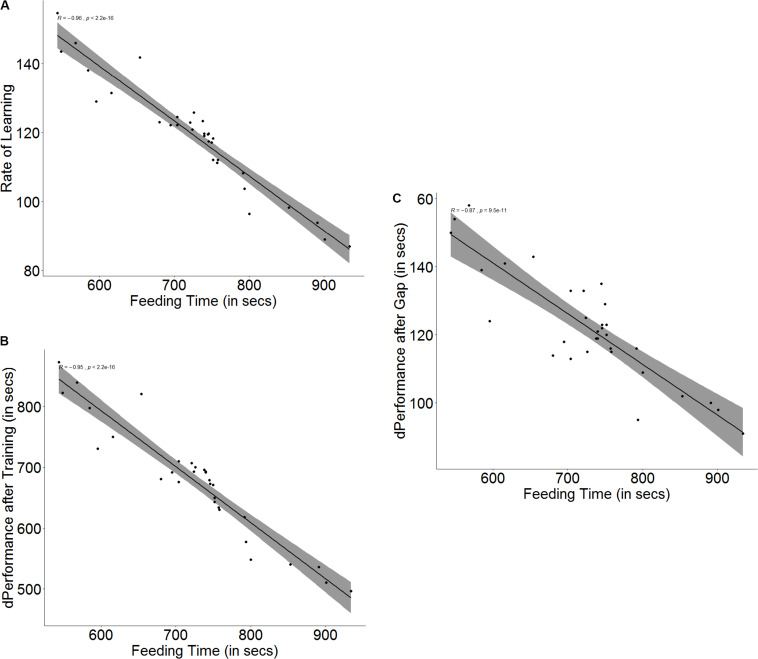
Scatter plot with regression line showing correlation between **(A)** feeding time and slope of learning curve, indicating rate of learning, **(B)** difference in performance after training, and **(C)** difference in performance after a 3-days gap.

The baseline ability for spatial navigation is a predictor for how fast a fish is able to learn (*r* = -0.96, *p* < 0.001, Figure 4A) as well as how much improvement it shows in its performance after training (*r* = -0.97, *p* < 0.001, Figure 4B). Fish that have better spatial ability show a more significant improvement in performance (*U* = 252.5, *p* < 0.001, *n*_1_ = 16, *n*_2_ = 16) and also a steeper learning curve (*U* = 253, *p* < 0.001, *n*_1_ = 16, *n*_2_ = 16) than fish that have poorer spatial navigation skills. Better spatial ability also resulted in poorer retention of memory (*r* = -0.87, *p* < 0.001, Figure 4C) when the fish were tested after the 3-day gap, since fish with better spatial skills showed greater difference in performance between the last training trial and the test trial (*U* = 199.5, *p* = 0.007, *n*_1_ = 16, *n*_2_ = 16) (see Supplementary Figure S4).

### Effect of Sex

**FIGURE 5:**
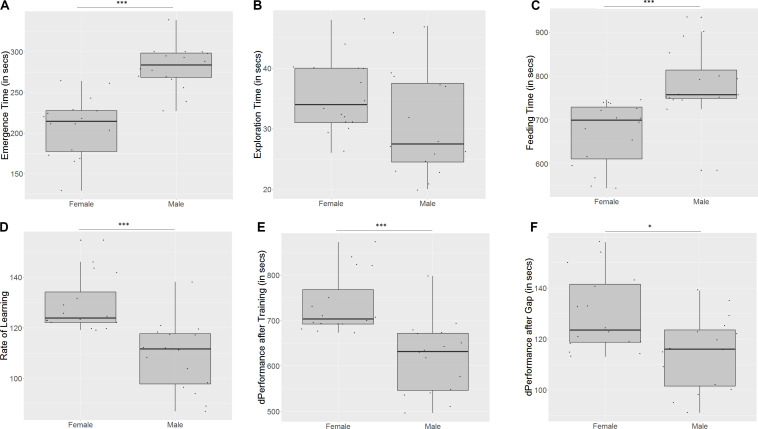
Females and males differ in **(A)** emergence time, **(B)** exploration time, **(C)** feeding time, **(D)** slope of learning curve, **(E)** difference in performance after training, and **(F)** difference in performance after a 3-day gap. “^∗^” indicates significant difference with *p* < 0.05, and “^∗∗∗^” indicates significant difference with *p* < 0.01.

Males took longer to emerge from the refuge chamber (*U* = 9.5, *p* < 0.001, *n*_1_ = 16, *n*_2_ = 16, Figure 5A) on the first trial, indicating that females are bolder than males. There was no significant difference in the time that males and females took to cross the exploration zone (*U* = 179, *p* = 0.06, *n*_1_ = 16, *n*_2_ = 16, Figure 5B), although males seemed more inclined to explore. Males also took longer to reach the food on the first day (*U* = 19.5, *p* < 0.001, *n*_1_ = 16, *n*_2_ = 16, Figure 5C), indicating poorer spatial navigation skills than females. Females showed a greater improvement in performance than males (*U* = 236, *p* < 0.001, *n*_1_ = 16, *n*_2_ = 16, Figure 5D) on the last training trial. They also displayed a steeper incline in the slope of the learning curve (*U* = 240, *p* < 0.001, *n*_1_ = 16, *n*_2_ = 16, Figure 5E), indicating that they learn more quickly than males. However, females show a greater difference in performance between the last training trial and the test trial (*U* = 187, *p* = 0.03, *n*_1_ = 16, *n*_2_ = 16, Figure 5F), implying that they have worse retention of memory than males.

### Effect of Predator

**FIGURE 6:**
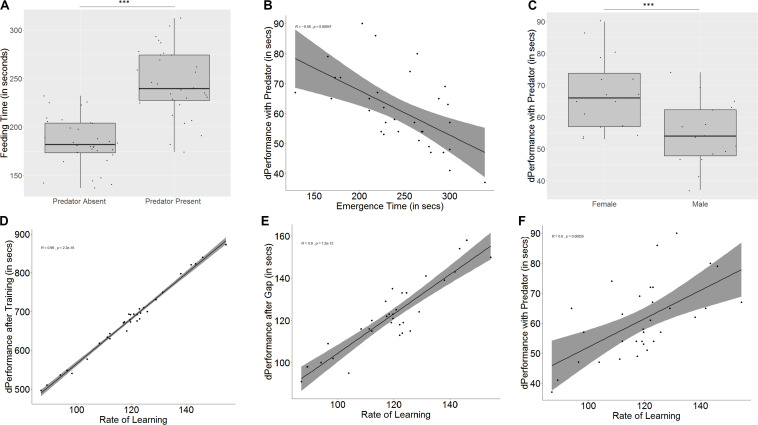
Role of predation on feeding latencies, performance, memory, and learning. **(A)** Time taken to feed increases in the presence of a predator; **(B)** difference in performance in the presence of a predator is affected by emergence time, indicating that bolder fish show greater difference in behavior; **(C)** males and females are affected differently in the presence of a predator, with females showing greater difference in performance; **(D)** rate of learning, denoted by slope of the learning curve affects quality of learning (measured as difference in performance after training); **(E)** slope of learning curve also affects retention of memory; and **(F)** slope of learning curve significantly affects differences in performance in the presence and absence of a predator. “***” indicates significant difference with *p* < 0.001.

The presence of a predator has a significant effect on the emergence time (*W* = 72, *p* < 0.001, *n* = 32) as well as the time taken to feed after crossing the maze (*W* = 0, *p* < 0.001, *n* = 32, Figure 6A), both of which increase, but not on the exploration time (*W* = 155, *p* = 0.11, *n* = 32), as compared to the same measures from the test trial without a predator.

The emergence time on day 1, taken as a measure for boldness, affects how much difference is seen in the time taken for emergence (*r* = -0.35, *p* = 0.04) and feeding (*r* = -0.58, *p* < 0.001, Figure 6B) between the two test trials, but the same trend was not seen for exploration time (*r* = -0.09, *p* = 0.59). In spite of the correlation, a significant difference between bold and shy fish was only seen in the difference between feeding times (*U* = 192, *p* = 0.02, *n*_1_ = 16, *n*_2_ = 16), where bolder fish took longer in the presence of the predator, and not for emergence time (*U* = 162, *p* = 0.21, *n*_1_ = 16, *n*_2_ = 16) or exploration time (*U* = 139, *p* = 0.69, *n*_1_ = 16, *n*_2_ = 16) (see Supplementary Figure S5).

In the presence of a predator, both males and females show similar emergence (*U* = 175.5, *p* = 0.08, *n*_1_ = 16, *n*_2_ = 16) and exploration times (*U* = 132.5, *p* = 0.88, *n*_1_ = 16, *n*_2_ = 16) to the test trial without a predator. However, females show less difference in feeding time than males (*U* = 202, *p* = 0.006, *n*_1_ = 16, *n*_2_ = 16, Figure 6C).

### Effect of Learning

Some fish learn faster (*U* = 0.5, *p* < 0.001, *n*_1_ = 16, *n*_2_ = 16, groups formed by ranking slope of learning curve) and better (*U* = 0, *p* < 0.001, *n*_1_ = 16, *n*_2_ = 16, groups formed by ranking difference in performance after training) than other fish. There is also a correlation between the rate and quality of learning (*r* = 0.99, *p* < 0.001). Fish that learn faster also show a greater improvement in performance than fish that are slower learners (*U* = 10.5, *p* < 0.001, *n*_1_ = 16, *n*_2_ = 16, Figure 6D), and fish that are better learners (i.e., show greater difference in performance after training) also learn faster (*U* = 6, *p* < 0.001, *n*_1_ = 16, *n*_2_ = 16). Both rate of learning (*r* = 0.9, *p* < 0.001, Figure 6E) as well as improvement in performance (*r* = 0.92, *p* < 0.001) have an effect on memory. Fish that learn faster and better (*U* = 51.5, *p* = 0.004, *n*_1_ = 16, *n*_2_ = 16, based on learning curve; *U* = 46.5, *p* = 0.002, *n*_1_ = 16, *n*_2_ = 16, based on performance difference) show greater difference in performance between the last training trial and the test trial, indicating that they have poorer memory.

Learning also affects difference in performance in the presence of a predator (*r* = 0.60, *p* < 0.001, based on learning curve, Figure 6F; *r* = 0.58, *p* < 0.001, based on difference in performance), and fish that learn faster and better also show less difference in performance in the presence of a predator (*U* = 53.5, *p* = 0.005, *n*_1_ = 16, *n*_2_ = 16; *U* = 256, *p* < 0.001, *n*_1_ = 16, *n*_2_ = 16).

## Discussion

Our study shows, that overall, fish showed a clear decline in emergence time, exploration time, and feeding latency over trials, pointing at an improvement in performance with time, which is indicative of learning or habituation. After the 3-day gap in training, there is an increase in the time taken to emerge from the shelter, explore, and then feed, but it is still lesser than that displayed by naive fish on the first day, clearly indicating that the fish retain some memory of the spatial task. It has also been shown that personality traits such as boldness, exploration, and spatial navigation ability affect each other (Mamuneas et al., 2015), as well as cognitive measures such as learning and memory (White et al., 2017). The presence of a predator results in greater time taken to emerge as well as to feed, and the extent of the difference depends on the personality of the fish. Males and females differ both in personality and cognitive traits, and although females emerge faster than males, males explore more readily and females navigate the maze more quickly. Females are also faster and better learners but show greater difference in performance after a break when compared with the last training trial, indicating poorer memory. Cognitive traits affect each other (e.g., learning is correlated to memory) as well as behavior in the presence of a predator. Fish that perform better cognitively are less affected in the presence of a predator.

Personality traits are known to be consistent and occur in similar patterns, resulting in behavioral syndromes (Bell, 2007; Conrad et al., 2011; Sih et al., 2012), which have significant ecological implications. These personality syndromes also affect cognitive traits, although the extent to which cognition is affected shows variation across traits (Sih and Del Giudice, 2012). Studies in guinea pigs have even shown that not all personality traits affect cognition in the same way and that there are variations in the way cognitive traits affect each other as well as personality (Guenther and Brust, 2017). In other species of fish, such as a guppies and mormyrid species, spatial learning has been shown to be affected by various aspects of personality such as boldness and exploration (Burns and Rodd, 2008; Kareklas et al., 2017). Personality (Moretz et al., 2007; Martins and Bhat, 2014; Roy et al., 2017; Roy and Bhat, 2018b) and spatial cognition (Arthur and Levin, 2001; Spence et al., 2011) in zebrafish have been characterized and studied, but there have been no forays into deciphering the relationship between the two aspects of behavior. However, recent studies have been performed in multiple taxa, aimed at distinguishing the underlying correlation between personality and cognition (Carter et al., 2014; Bousquet et al., 2015; Guillette et al., 2017). Our study examined correlations between personality and spatial learning in wild zebrafish and whether personality traits remain consistent over time and context.

### Personality Correlates and Effects on Cognition

Boldness was shown to have an effect on other aspects of personality such as exploration and spatial navigation ability, as well as on cognitive measures such as learning and memory. Bolder fish were quicker to explore as well as had better navigation skills in a spatial task. In zebrafish, this behavioral syndrome has been noted in many studies (Sutrisno et al., 2011; Norton and Bally-Cuif, 2012) and might lead to improved spatial navigation abilities as well. Bolder fish also had better rates of learning and also showed greater improvement in their performance at the end of the training trial, which has been shown in other species such as mallards (Bousquet et al., 2015), lizards (Carazo et al., 2014), and even in a mormyrid fish (Kareklas et al., 2017) but was hitherto unreported in zebrafish. Memory, however, was shown to be poor in bolder fish. Since fish with greater boldness and exploration tendencies are more likely to venture into new habitats and hence need better cognitive abilities to assess and perform well in them. However, they are less likely to remain in the same area for longer lengths of time, and therefore, they do not have the need for improved memory in order to adapt and perform well (White et al., 2017).

### Sex Differences in Personality and Cognition

Sex has been shown to have an effect on personality as well as learning and memory. Although females were bolder than males in terms of the time taken to emerge from a refuge chamber, they were less ready to explore and navigate the maze. Some studies in zebrafish have indicated that males are bolder than females (Roy and Bhat, 2018b). However, other studies have demonstrated that there is no difference between males and females in terms of boldness (Way et al., 2015), and any difference that is present is not consistent across populations and contexts in zebrafish (Roy and Bhat, 2018a). In other fish, such as guppies *Poecilia reticulata* (King et al., 2013) and *Brachyrhaphis episcopi* (Archard and Braithwaite, 2011), females tend to be bolder, but this effect is more pronounced in individuals from high-risk habitats, indicating that sex-dependent differences largely depend on ecological factors.

Learning ability and retention of memory has also been shown to differ in males and females, with males showing better learning and females showing better memory. In guppies, spatial learning in a complex maze is performed only by males (Lucon-Xiccato and Bisazza, 2017b) and not by females, which reflects the different strategies of the sexes in their natural habitat, with the males being farther ranging and the females remaining in restricted locations. The males in our study also showed a greater tendency to explore. However, they took longer than females to emerge from a refuge and to navigate the maze and feed on the first day, and this could result in the females showing more efficient learning in the maze. However, as with boldness, individuals that explore more will remain in the same place for less time, making it futile to have a good memory, which might be advantageous for females. Indeed, this also appears to be highly habitat and context specific – in some populations of pond snails (*Lymnaea stagnalis*), when isolated, slower explorers were found to form longer memories, while in groups, this correlation was not found (Dalesman, 2018).

### Predator Presence and Its Effect

The presence of a predator affected emergence as well as feeding latency in all individuals. Bolder individuals, however, were less affected and emerged more quickly from the refuge chamber than shy individuals. Change in personality-associated behavior due to the presence of a predator has been shown in several fish species, such as carps, perches, and rainbow trout (Ioannou et al., 2008; Magnhagen and Borcherding, 2008; Thomson et al., 2012). However, in terms of feeding latency, bolder fish took longer to feed in the presence of a predator, which was a result of them performing far more predator inspections than shy fish, resulting in a delay in the time taken to feed (Dugatkin et al., 2005). Females showed less difference in performance in the presence of the predator, which could be because females are inherently bolder than males in other contexts.

### Effect of Learning

Although learning itself is affected by personality traits, the converse could also hold true. Fish that perform better at learning might be cognitively superior to the fish that perform poorly, and this allows them to take more risks, which results in them being bold. This could also lead to them being more adept at weighing the danger posed by a confined predator, and hence, they are less likely to show a difference in behavior when under threat.

In a rapidly changing environment, fish that are more willing to explore a novel environment are more likely to thrive. Bolder, proactive individuals are likely to take risks to explore novel environments that might lead to greater reproductive success than reactive individuals that are unwilling to take risks and generally shy (Carere and Locurto, 2011; Griffin et al., 2015). Being better at learning and other cognitive functions ensures better adaptability in a new or changing environment. Studies have shown that bolder fish do perform better in rapidly changing environments or if they are translocated to a completely new habitat by natural processes such as water flow (Wright et al., 2003), which might be due to better cognitive abilities that accompany boldness. Indeed, it has been observed for the Atlantic cod (*Gadus morhua*) that personality traits can influence movements and responses to changes in seawater temperature where proactive individuals are more likely to be able to expand their home ranges than reactive ones (Villegas-Ríos et al., 2018). However, studies in other fish species, such as sticklebacks (Jolles et al., 2019) and rainbow trout (de Lourdes Ruiz-Gomez et al., 2011), have shown that shy or reactive individuals show sensitivity to changes and perform better in a new environment, as bolder fish show routine formation and are unable to adapt rapidly. It is likely that other factors such as physiological and life history traits may also be involved in responses to environment (Mathot et al., 2012; Villegas-Ríos et al., 2018). These contradictory results clearly indicate that further studies are necessary to understand the complex interplay of personality and response to environmental changes.

Although, so far, studies have shown that, for most species, there is some correlation between boldness and cognitive ability (Dougherty and Guillette, 2018), the underlying causes are not really known. Brain anatomy and physiology studies have shown that larger brain sizes are related with both bolder personalities (Kotrschal et al., 2014) and better cognitive abilities (Lucon-Xiccato and Bisazza, 2017a) in guppies. While it is not completely understood which parts of the brain specifically govern personality, studies in this direction could shed light upon why there is a correlation between personality and cognition. It also remains to be seen if this relationship persists across different populations of a species and whether it is modified by the local environment. It would also be interesting to decipher the directionality of this correlation and shed light on whether personality is affected by cognitive ability, or vice versa.

## Data Availability Statement

The raw data supporting the conclusions of this article will be made available by the authors, without undue reservation, to any qualified researcher.

## Ethics Statement

The animal study was reviewed and approved by the Committee for the Purpose of Control and Supervision of Experiments on Animals (CPCSEA), Government of India Institutional Animal Ethics Committee’s (IAEC) and guidelines of Indian Institute of Science Education and Research (IISER) Kolkata.

## Author Contributions

AB and DD conceived the goals and the design of the study, wrote and edited the final version of manuscript. DD conducted the experimental assays, analyzed the data, performed the statistical analyses, and wrote the first draft of the manuscript. Both authors read and approved the submitted version.

## Conflict of Interest

The authors declare that the research was conducted in the absence of any commercial or financial relationships that could be construed as a potential conflict of interest.
