# Supplementary material for: Bolder and Brighter? Exploring Correlations Between Personality and Cognitive Abilities Among Individuals Within a Population of Wild Zebrafish, Danio rerio
Source: Front Behav Neurosci. 2020 Aug 12;14:138. doi: 10.3389/fnbeh.2020.00138 (PMC7438763; doi:10.3389/fnbeh.2020.00138)
Supplement: Supplementary file 1 [file Data_Sheet_1.docx]

**Supplementary Figures**


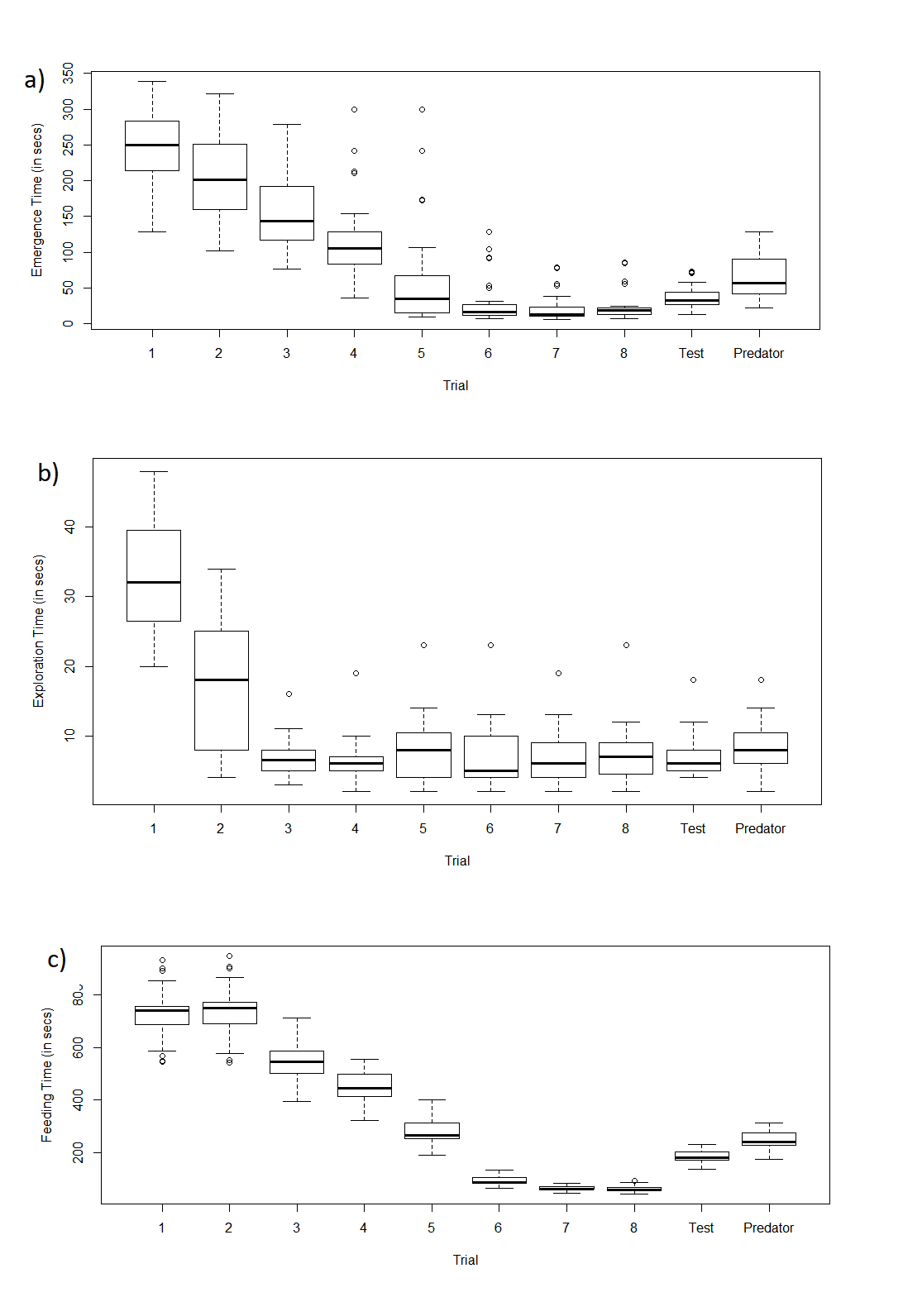


Figure S1. Emergence time (a), exploration time (b) and feeding time (c) over trials 1 to 8, followed by the test trial and the trial in the presence of a predator. Latencies typically declined over trials, with slight increase after a three day gap and increased in the presence of a predator.


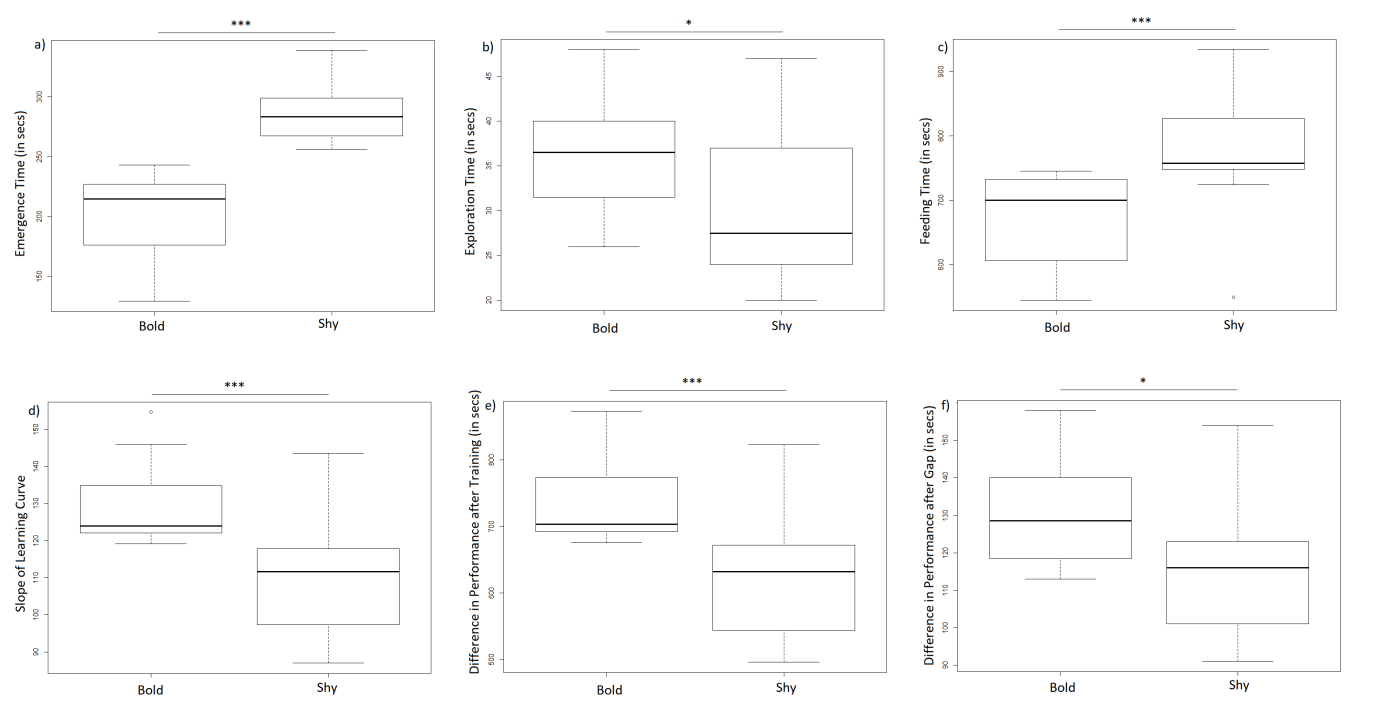


Figure S2. Personality and cognition differences between bold and shy fish. Bolder fish emerge faster from the shelter (a), take longer to cross the exploration zone (b) and inherently take less time to navigate the maze (c). Bolder fish also show faster and better learning, but poorer memory than shy fish. ‘*’ indicates significant difference with p<0.05 and ‘***’ indicates significant difference with p<0.01.


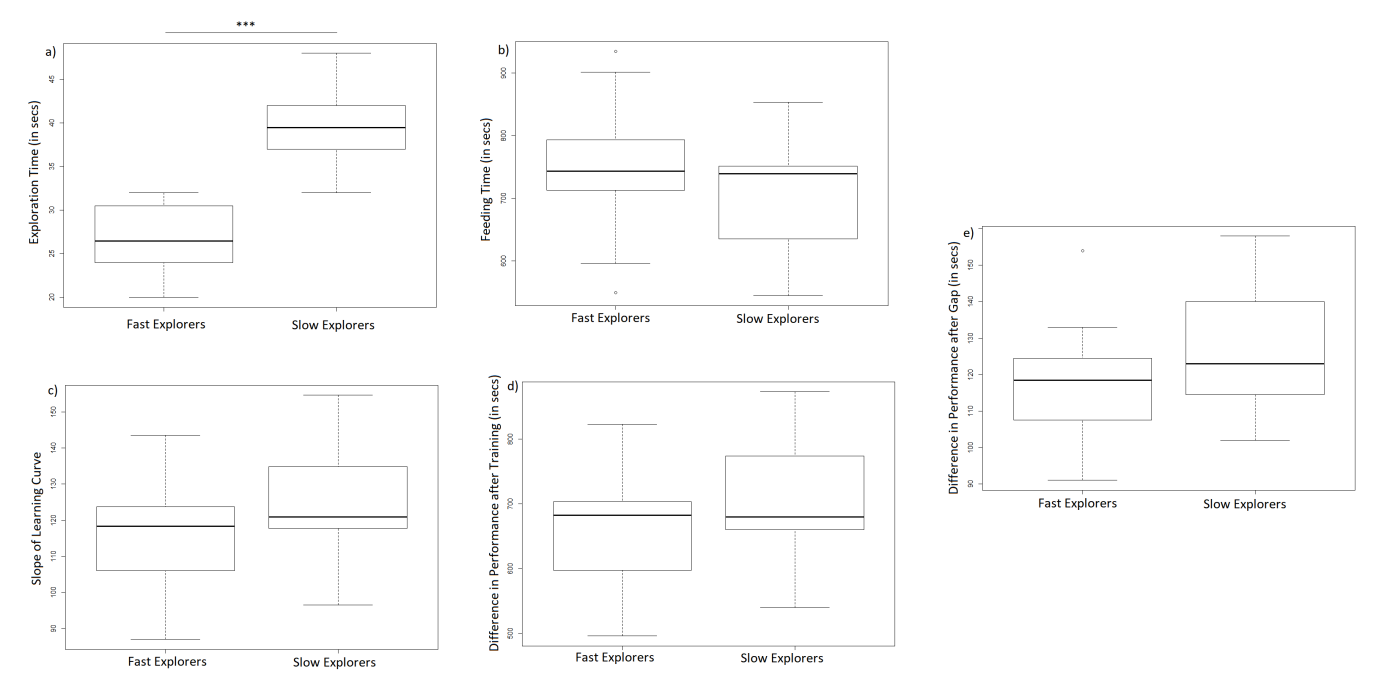


Figure S3. Personality and cognition differences between fast and slow explorers. Fish that explore faster cross the exploration zone faster than fish that are slow explorers (a). There is no difference between fast and slow explorers in spatial ability (b), speed of learning (c), improvement in performance (d) or retention of memory (e). ‘*’ indicates significant difference with p<0.05 and ‘***’ indicates significant difference with p<0.01.


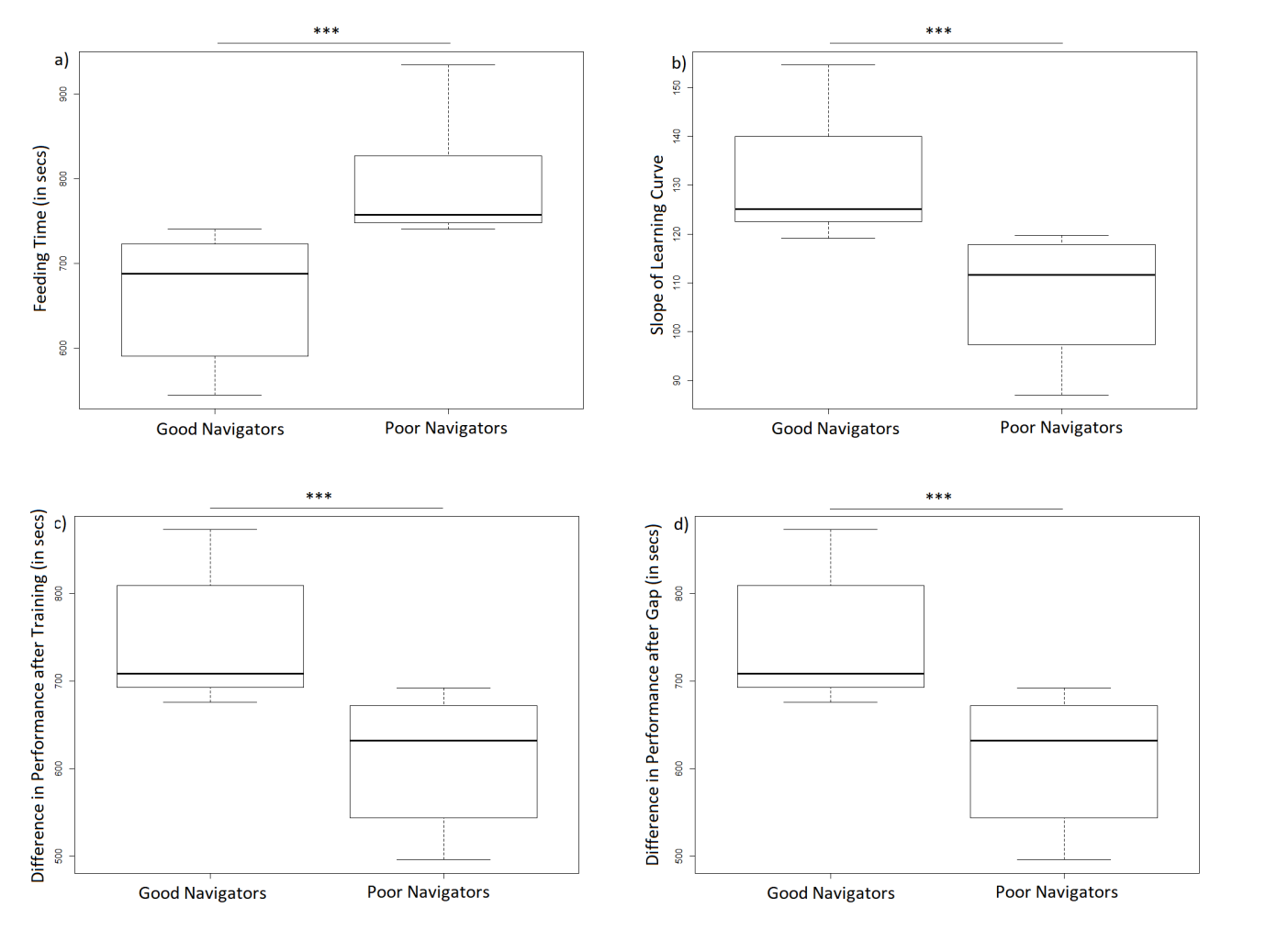
Figure S4. Differences spatial ability or navigation skills and its effect on cognition. Fish that are better navigators show perform better in the spatial task without any training than poor navigators (a). Good navigators also show greater slope in their learning curves (b), greater improvement in performance after training (c), and poorer retention of memory after a gap (d). ‘*’ indicates significant difference with p<0.05 and ‘***’ indicates significant difference with p<0.01.


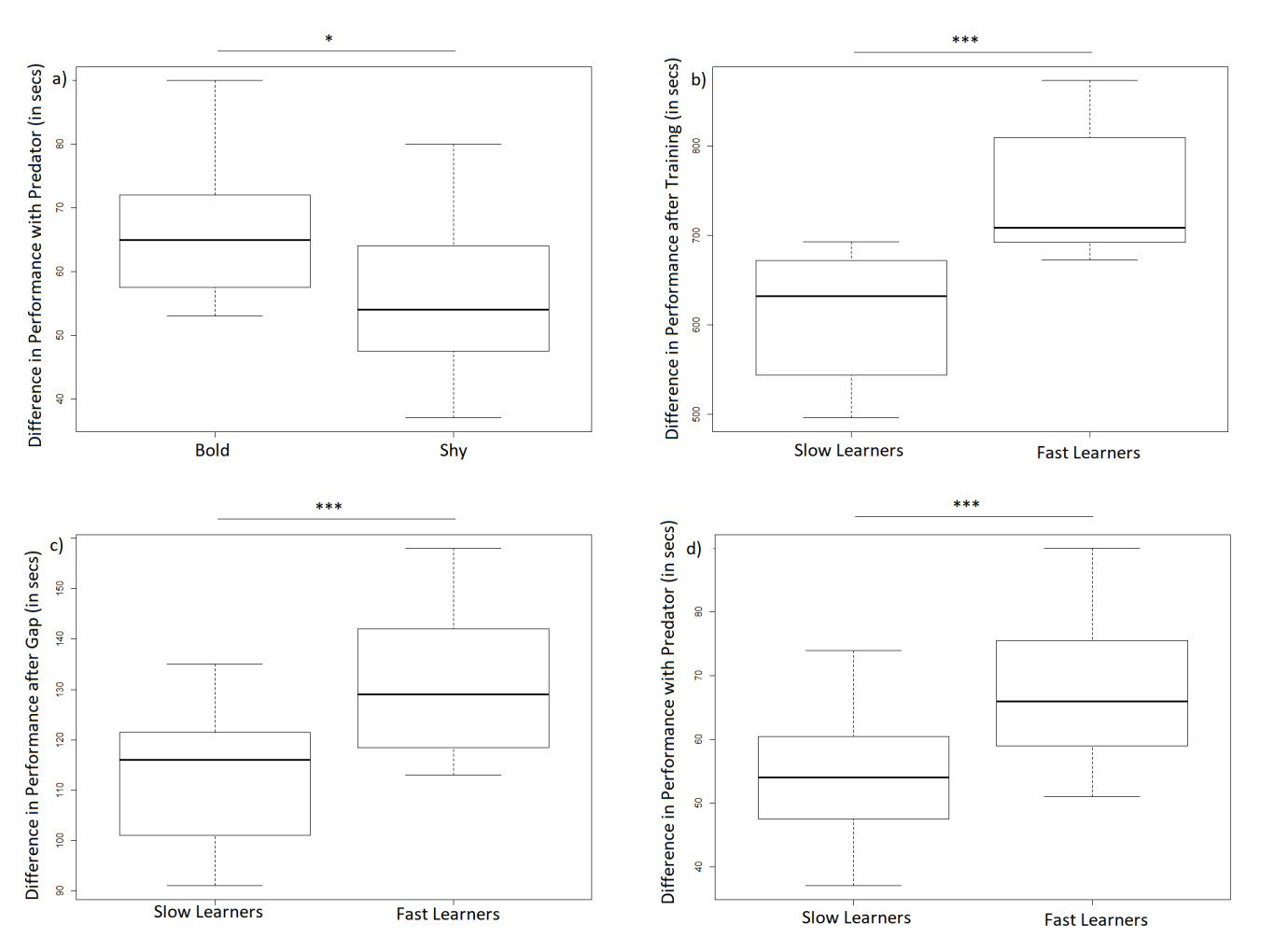


Figure S5. Bolder fish show greater difference in performance in the presence of a predator (a). Faster learners show greater improvement of performance after training (b), less retention of memory after a gap (c), and less difference in behaviour in the presence of a predator (d). ‘*’ indicates significant difference with p<0.05 and ‘***’ indicates significant difference with p<0.01.
