# Supplementary material for: Bolder and Brighter? Exploring Correlations Between Personality and Cognitive Abilities Among Individuals Within a Population of Wild Zebrafish, Danio rerio
Source: Front Behav Neurosci. 2020 Aug 12;14:138. doi: 10.3389/fnbeh.2020.00138 (PMC7438763; doi:10.3389/fnbeh.2020.00138)
Supplement: Supplementary file 2 [file Table_1.DOCX]

| ***Supplementary Table S1***  Mean values and standard error for observations on trials used for comparison. | **Trial 2** | | **Trial 8** | | **Test Trial** | | **Trial with Predator** | |
| --- | --- | --- | --- | --- | --- | --- | --- | --- |
|  | Mean | Std. Error | Mean | Std. Error | Mean | Std. Error | Mean | Std. Error |
| **Emergence Time** | 202.59 | 10.57 | 23.5 | 3.42 | 36.38 | 2.84 | 67.78 | 5.35 |
| **Exploration Time** | 17.5 | 1.56 | 7.16 | 0.68 | 6.91 | 0.51 | 8.25 | 0.6 |
| **Feeding Time** | 738.59 | 17.78 | 61.91 | 2.31 | 184.19 | 4.44 | 245.28 | 6.02 |

| ***Supplementary Table S2***  Mean values and standard error of measures for groups used in pairwise comparisons. | | | **Emergence Time** | **Exploration Time** | **Feeding Time** | **Slope of Learning Curve** | **Difference in Performance after Training** | **Difference in Performance after Gap** | **Difference in Feeding Time in Presence of Predator** | **Difference in Emergence Time in Presence of Predator** | **Difference in Exploration Time in Presence of Predator** |
| --- | --- | --- | --- | --- | --- | --- | --- | --- | --- | --- | --- |
| **Sex** | *Female* | Mean | 207.88 | 35.56 | 670.06 | 129.29 | 734.88 | 129.88 | 67.44 | 37.25 | 1.69 |
|  |  | Std. Error | 8.88 | 1.47 | 17.52 | 2.67 | 15.97 | 3.64 | 2.81 | 4.96 | 0.66 |
|  | *Male* | Mean | 281 | 31 | 783.38 | 108.97 | 618.5 | 114.69 | 54.75 | 25.56 | 1 |
|  |  | Std. Error | 6.54 | 2.11 | 20.12 | 3.28 | 19.32 | 3.48 | 2.45 | 3.99 | 0.81 |
| **Boldness** | *Bold* | Mean | 204.18 | 36.56 | 672.25 | 128.94 | 733.69 | 129.69 | 66.38 | 36.13 | 1.63 |
|  |  | Std. Error | 7.74 | 1.52 | 16.64 | 2.58 | 15.41 | 3.35 | 2.7 | 5.11 | 0.48 |
|  | *Shy* | Mean | 284.69 | 30 | 781.19 | 109.32 | 619.69 | 114.88 | 55.81 | 26.69 | 1.06 |
|  |  | Std. Error | 5.13 | 1.9 | 21.56 | 3.48 | 20.2 | 3.81 | 2.86 | 4 | 0.92 |
| **Exploration** | *Fast Explorers* | Mean | – | 26.88 | 751.38 | 114.41 | 652.25 | 117.44 | 59.06 | – | – |
|  |  | Std. Error | – | 0.93 | 24.35 | 3.68 | 21.17 | 3.88 | 3.32 | – | – |
|  | *Slow Explorers* | Mean | – | 39.69 | 702.06 | 123.85 | 701.13 | 127.13 | 63.13 | – | – |
|  |  | Std. Error | – | 1.13 | 21.07 | 3.8 | 23 | 3.82 | 2.72 | – | – |
| **Navigation** | *Good Navigators* | Mean | – | – | 659 | 130.51 | 742.75 | 131.13 | 67.63 | – | – |
|  |  | Std. Error | – | – | 17.24 | 2.63 | 15.86 | 3.62 | 2.8 | – | – |
|  | *Bad Navigators* | Mean | – | – | 794.44 | 107.75 | 610.63 | 113.44 | 54.56 | – | – |
|  |  | Std. Error | – | – | 15.57 | 2.77 | 15.96 | 3.12 | 2.4 | – | – |
| **Improvement in Performance** | *Bad Learners* | Mean | – | – | – | 107.9 | 2.82 | 130.36 | 2.66 | – | – |
|  |  | Std. Error | – | – | – | 609.63 | 15.66 | 743.75 | 15.62 | – | – |
|  | *Good Learners* | Mean | – | – | – | 112.94 | 3.08 | 131.63 | 3.49 | – | – |
|  |  | Std. Error | – | – | – | 743.75 | 15.62 | 609.63 | 15.66 | – | – |
| **Rate of Learning** | *Slow Learners* | Mean | – | – | – | 107.71 | 2.76 | 130.55 | 2.62 | – | – |
|  |  | Std. Error | – | – | – | 611.88 | 16.3 | 741.5 | 16.15 | – | – |
|  | *Fast Learners* | Mean | – | – | – | 113.19 | 3.08 | 131.38 | 3.57 | – | – |
|  |  | Std. Error | – | – | – | 54.63 | 2.4 | 67.56 | 2.82 | – | – |
